# Identification of a Glycosyltransferase-Encoding Gene (*EuGT8*) from *Eucommia ulmoides* That Catalyzes the Glycosylation of Pinoresinol to Pinoresinol Diglucoside

**DOI:** 10.3390/life16040622

**Published:** 2026-04-08

**Authors:** Xian Gong, Lijun Qin

**Affiliations:** 1 Institute of Agro-Bioengineering, College of Life Science, Guizhou University, Guiyang 550025, China; sgong@gzu.edu.cn; 2 The Key Laboratory of Plant Resources Conservation and Germplasm Innovation in the Mountainous Region (Ministry of Education), Xueshi Road, Guiyang 550025, China

**Keywords:** *E. ulmoides*, PDG, *EuGT8* gene, prokaryotic expression, transcriptional analysis, metabolomic analyses

## Abstract

Pinoresinol diglucoside (PDG), one of the major lignans isolated from *E. ulmoides* Oliver bark, has various pharmacological functions, including antihypertension and prevention of osteoporosis. However, the glycosyltransferase-encoding gene (*GT*) involved in regulating the glycosylation of pinoresinol to form PDG has not been reported in *E. ulmoides*. In this study, we screened and cloned the *EuGT8* gene from *E. ulmoides* based on our transcriptome data. The expression pattern of the *EuGT8* gene exhibited a strong positive correlation with dynamic changes in the PDG contents in three different organs of *E. ulmoides*. The expression level of the *EuGT8* gene and PDG content were significantly decreased in asODN-*EuGT8*-treated shoot tips in comparison with the control group. Prokaryotic expression of the *EuGT8* gene revealed that the purified EuGT8 protein could catalyze the conversion of pinoresinol into PDG. In addition, we performed transcriptional and metabolomic analyses to compare the differences between transgenic *Arabidopsis* and WT plants. A total of 1799 DEGs and 294 DEMs were identified in transgenic and WT plants. KEGG enrichment analysis showed that the DEGs were mainly enriched in phenylpropanoid biosynthesis, secondary metabolite biosynthesis, and starch/sucrose metabolism pathways. The DEMs were mainly enriched in ABC transporters, aminoacyl-tRNA biosynthesis, biosynthesis of amino acids, phenylpropanoid biosynthesis, and flavone and flavonol biosynthesis pathways. Correlation analysis between DEGs and DEMs identified a total of 231 DEGs associated with 38 DEMs, which were mainly distributed in multiple metabolic pathways. This finding provides both theoretical insights and genetic resources for breeding high-PDG *E. ulmoides* varieties, facilitating marker-assisted selection (MAS) and promoting sustainable *E. ulmoides* production in Guizhou.

## 1. Introduction

*Eucommia ulmoides* Oliv. (also called “Du Zhong”) is a deciduous tree species endemic to China, belonging to the family Eucommiaceae and genus Eucommia. Its leaves, stem, bark, and flowers have long been used as medicinal materials [1]. Numerous studies showed that the aerial parts of *E. ulmoides* contained numerous bioactive compounds, including lignans, iridoids, flavonoids, and phenylpropanoids [2,3,4,5]. Among them, lignans constitute a group of diphenolic compounds, including pinoresinol, lariciresinol, secoisoariciresinol, syringaresinol, and sesamin, which exhibit a broad spectrum of biological activities such as antioxidant, antihypertensive, anticancer, antiviral, estrogenic, and insecticidal effects [6,7].

The plant lignans are mainly biosynthesized via the phenylpropanoid pathway. This pathway starts with the conversion of phenylalanine, which is sequentially catalyzed by phenylalanine ammonia-lyase (PAL), cinnamate-4-hydroxylase (C4H), and 4-coumarate-CoA ligase (4CL) to form 4-coumaroyl-CoA [8]. As a key precursor, 4-coumaroyl-CoA is further modified by enzymes including caffeic acid O-methyltransferase (COMT) and ferulate-5-hydroxylase (F5H), yielding substituted cinnamoyl-CoA derivatives such as feruloyl-CoA and sinapoyl-CoA. These derivatives contribute substantially to the structural diversity of downstream lignans. They are then reduced by cinnamoyl-CoA reductase (CCR) and cinnamyl alcohol dehydrogenase (CAD) to produce monolignols (e.g., coniferyl alcohol, sinapyl alcohol) [9]. Next, oxidative coupling of monolignols catalyzed by laccases (LACs) or class III peroxidases (PODs) leads to the formation of lignan dimers (e.g., pinoresinol, sesamin). Following dimerization, glycosyltransferases (GTs) catalyze the transfer of sugar moieties (e.g., glucose, rhamnose) to the hydroxyl groups of lignans, resulting in lignan glycosides [10]. This glycosylation modification improves the solubility of lignans to facilitate their intracellular transport and vacuolar storage, while also reducing their potential cytotoxicity [11].

Glycosyltransferases (GTs) belong to a diverse family of enzymes that catalyze the transfer of sugar moieties from activated donor sugars to specific acceptors, thus playing a crucial role in various biological processes [12,13,14]. Numerous studies have reported that GT family genes play a crucial role in the glycosylation modification of lignans [15,16]. For example, Chen et al. (2021) revealed that tandem *UGT71B5* genes were involved in catalyzing lignan glycosylation in *Isatis indigotica* with substrate promiscuity [17]. The *IiUGT71B5a* gene participated in generating both pinoresinol monoglycoside and diglycoside, and the *IiUGT71B5b* gene only produced monoglycoside. In *Linum usitatissimum*, the LuUGT74S1 was identified as a key enzyme controlling the formation of secoisolariciresinol diglucoside [18]. In addition, GTs have also been reported to participate in the glycosylation of lignan precursors. For example, the *AtUGT72E2* and *AtUGT72E3* genes from *Arabidopsis* have been demonstrated to glycosylate phenylpropanoid alcohol precursors of lignan biosynthesis (sinapyl- and coniferyl-alcohol) [19]. The UGT74S1 is the key player in controlling secoisolariciresinol diglucoside (SDG) formation in flax. Previous studies revealed that PDG, one of the major lignans isolated from *E. ulmoides* Oliver bark, has various pharmacological functions, including antihypertension and prevention of osteoporosis [1,20,21]. However, the *GT* gene that is involved in regulating the glycosylation of pinoresinol to form PDG has not been reported in *E. ulmoides*.

In this study, we initially identified and cloned an *EuGT8* gene on the basis of our laboratory’s transcriptome data. We also investigated the expression patterns of the *EuGT8* gene and the dynamic changes in PDG contents in three different organs of *E. ulmoides*. The prokaryotic expression was applied to verify whether the *EuGT8* gene was involved in regulating the glycosylation of pinoresinol to form PDG. In addition, integrated transcriptomic and metabolomic analyses revealed that the *EuGT8* gene played essential roles in regulating the biosynthesis of some important secondary metabolites, particularly phenylpropanoids and flavonoids. Our findings provide both theoretical insights and genetic resources for breeding high-PDG *E. ulmoides* varieties, facilitating marker-assisted selection (MAS) and promoting sustainable *E. ulmoides* production in Guizhou.

## 2. Materials and Methods

### 2.1. Plant Materials and Growth Conditions

The eight-year-old *E. ulmoides* plants were cultivated in the Eucommia resource nursery of the key laboratory of mountain plant resources conservation and germplasm innovation, Ministry of Education, Guizhou University. All test materials in the garden were managed per standardized cultivation protocols under natural ambient conditions with sufficient sunlight, a relative humidity of 60–75%, and daily temperatures ranging from 18 °C to 30 °C. Plants were irrigated regularly to maintain appropriate soil moisture, and a balanced compound fertilizer (N:P:K = 15:15:15) was applied monthly during the growing season to ensure a consistent water and nutrient supply. The leaves, stems, and roots were collected and immediately stored in liquid nitrogen for RT-qPCR and determining PDG contents.

The seeds of *Arabidopsis* ecotype Columbia-0 (Col-0) were germinated and grown in a growth chamber for 40 days under the following environmental regimes, including a photoperiod of 16 h light (25 °C)/8 h dark (20 °C), 60% relative humidity, and a photosynthetic photon flux density of 200 μmol m^−2^ s^−1^, which were used for genetic transformation. Transgenic *Arabidopsis* plants were used for transcriptomic and metabolomic analyses [22].

### 2.2. Expression and Purification of EuGT8

The coding sequence of *EuGT8* was cloned into the pET-32a(+) expression vector and then transformed into the *E. coli* BL21 strain [23]. When the OD600 value reached 0.5–0.7, protein expression was induced with 1 mM isopropyl β-D-1-thiogalactopyranoside (IPTG). Following incubation at 16 °C with shaking at 200 rpm for 10–16 h, bacterial cells were collected by centrifugation at 4 °C. Cells were resuspended in 30 mL 1× Phosphate-Buffered Saline (PBS), supplemented with 30 μL β-mercaptoethanol and 300 μL phenylmethylsulfonyl fluoride (PMSF). Cell lysis was performed by ultrasonication in an ice bath. After centrifugation at 12,000 rpm for 15 min at 4 °C, the supernatant containing soluble protein was collected. The his-tagged recombinant protein was further purified via Ni-NTA affinity chromatography using Bio-Scale Mini Profinity IMAC cartridges (BIO-RAD, Hercules, CA, USA) [24]. Purified protein samples were analyzed by SDS-PAGE. Eluates with high purity were selected for dialysis and concentration. Selected eluates were dialyzed overnight at 4 °C against 1× PBS containing 10% glycerol, pH 7.4, until the target protein remained clear with no visible precipitation. The target protein was concentrated using an ultrafiltration tube at 4500 rpm and 4 °C to an appropriate concentration. The final product was analyzed by SDS-PAGE, and the protein concentration and yield were determined.

### 2.3. In Vitro Enzyme Activity Assay

Using pinoresinol as the substrate, UDP-glucose as the sugar donor, and the purified EuGT8 protein as the catalyst, the enzymatic reaction was conducted in a 50 μL volume of 100 mM phosphate buffer (pH 8.0), containing 2 mM sugar donor, 200 μM substrate, and 1 μg purified protein. The enzyme-free reaction mixture was pre-incubated at 37 °C for 10 min, followed by the addition of the purified protein and further incubation at 37 °C for 1 h. The reaction products were analyzed by liquid chromatography–tandem mass spectrometry (LC-MS/MS) [25].

### 2.4. Determination of the PDG Contents Using LC-MS/MS Analysis

The PDG contents were determined using an Ultra Performance Liquid Chromatography (UPLC) system coupled with Tandem Mass Spectrometry (MS/MS) (ExionLC^TM^ AD, SCIEX, Framingham, MA, USA) [26]. Liquid chromatography conditions were set as follows: Agilent SB-C18 column (1.8 μm, 2.1 mm × 100 mm); mobile phase: (A) ultrapure water supplemented with 0.1% formic acid, and (B) acetonitrile supplemented with 0.1% formic acid. The elution gradient was programmed as: 0.00 min, 5% B; linearly increased to 95% B within 9.00 min and held for 1 min; from 10.00 to 11.10 min, B was decreased back to 5% and re-equilibrated until 14 min. The optimized chromatographic conditions were as follows: flow rate of 0.35 mL/min, column temperature maintained at 40 °C, and injection volume of 2 μL. For mass spectrometry, the optimized parameters were set as: electrospray ionization (ESI) source temperature of 500 °C; ion spray voltage (IS) of 5500 V in positive ion mode and −4500 V in negative ion mode. Ion Source Gas I (GSI), Gas II (GSII), and Curtain Gas (CUR) were optimized to 50, 60, and 25 psi, respectively, with optimized collision-induced dissociation parameters suitable for protein detection. Multiple reaction monitoring (MRM) scans were conducted in QQQ mode using medium-pressure nitrogen as collision gas. For the three target compounds, the MRM parameters were optimized by automatic and manual tuning with reference standards under the above-mentioned UPLC-MS/MS conditions. For pinoresinol (MW = 358), the optimized MRM parameters in positive ion mode were: precursor ion m/z 357.1 [M+H]^+^, quantitative product ion m/z 151.0, DP = 80 V, and CE = 20 V, with a qualitative ion pair of m/z 357.1→136.0 (DP = 80 V, CE = 22 V) for confirmation. For pinoresinol monoglucoside (MW = 520), the optimized MRM parameters were: precursor ion m/z 519.2 [M+H]^+^, quantitative product ion m/z 357.1, DP = 85 V, and CE = 22 V, with a qualitative ion pair of m/z 519.2→151.0 (DP = 85 V, CE = 24 V). For pinoresinol diglucoside (PDG, MW = 682), the optimized MRM parameters were: precursor ion m/z 681.2 [M+H]^+^, quantitative product ion m/z 358.1, DP = 90 V, and CE = 23 V, with a qualitative ion pair of m/z 681.2→151.0 (DP = 90 V, CE = 28 V). All parameters were validated based on the MS/MS fragmentation spectra of the authentic standards, ensuring the accuracy and reliability of the quantitative analysis.

### 2.5. Subcellular Localization of EuGT8

The pCambia1301-35S::EuGT8::eGFP vector was constructed and transformed into *Agrobacterium tumefaciens* strain GV1301. The agrobacteria were infiltrated into the abaxial side of leaves from 4-week-old *Nicotiana benthamiana* plants [27]. Samples were taken 48–72 h post-infiltration. *Agrobacterium* containing pCambia1301-35S::eGFP was used as a negative control. Imaging was performed using a confocal laser scanning microscope (Nikon C2-ER, Nikon, Tokyo, Japan).

### 2.6. Antisense Oligonucleotides (asOND)

Soligo software (v. 2.2, https://sfold.wadsworth.org) was applied to design asOND as a potential antisense inhibitor targeting *EuGT8*, with sense oligonucleotides (sOND) serving as controls [28]. All oligonucleotides were subsequently synthesized by Sangon Biotech Bioengineering (Shanghai, China). The apical buds of *E. ulmoides* were soaked separately in aqueous sOND (30 OD) and asOND (30 OD) for 48 h at ambient temperature.

### 2.7. Quantitative Real-Time PCR (qRT-PCR)

Total RNA was extracted using the TransZol Plus RNA Kit (ER501, TransGen, Beijing, China). cDNA was synthesized via one-step reverse transcription (PrimeScript^TM^ 1st Strand cDNA Synthesis Kit, 6110A, TAKARA, Beijing, China) using 2 μg total RNA as template. Gene expression levels were detected by qRT-PCR [TB Green^®^ Premix Ex Taq^TM^ (Tli RNaseH Plus), RR420A, TAKARA, China; QuantStudio^TM^ 3, Applied Biosystems, Carlsbad, CA, USA] [29]. Each sample group had four biological replicates, and each biological replicate was assayed three times. Primer information is listed in Supplementary Table S1. Real-time PCR reactions were performed as follows: denaturation at 95 °C for 10 min, followed by 40 cycles of 95 °C for 30 s, 62 °C for 30 s. Following the final amplification cycle, a melting dissociation curve was generated to ensure the specificity of the primers and to confirm the uniqueness of the amplification product. The output data was determined following the 2^−∆∆CT^ method, and it is reported as fold changes in relative expression.

### 2.8. Generation of Transgenic Arabidopsis Plants

The full-length CDS of *EuGT8* was amplified and inserted into the pCambia2300-GFP vectors to generate pCambia2300-*EuGT8*-GFP vectors. After restriction enzyme digestion verification, the positive *EuGT8*-OE vectors were transformed into *Agrobacterium* GV3101 by electroporation. The transgenic *Arabidopsis* plants overexpressing the *EuGT8* gene were generated by the *Agrobacterium* floral dipping method [22]. Transgenic seedlings were screened on 1/2 MS solid medium containing 50 mg/L hygromycin. Resistant plants were further verified by genomic PCR. T3 homozygous transgenic lines with stable segregation were used for subsequent phenotypic and molecular analyses.

PCR amplification was performed in a 20 μL reaction system, with pre-denaturation at 94 °C for 5 min, followed by 35 cycles of 94 °C for 30 s, 58 °C for 30 s, and 72 °C for 1 min, with a final extension at 72 °C for 10 min.

### 2.9. Transcriptome Sequencing and Analysis

Total RNA was extracted from transgenic *Arabidopsis* and WT plants using TRIzol^®^ reagent (Invitrogen, Carlsbad, CA, USA). The mRNA was purified from 1 μg total RNA using oligo(dT) magnetic beads, fragmented, and then used to synthesize double-stranded cDNA. Library quality was assessed using an Agilent Bioanalyzer 4150. Finally, sequencing was performed on the Illumina Novaseq 6000/MGISEQ-T7 platform. Clean reads were aligned to the *Arabidopsis* reference genome (https://ftp.ensemblgenomes.ebi.ac.uk/pub/plants/release-51/gff3/arabidopsis_thaliana/, accessed on 10 April 2025) using HISAT2 software (http://daehwankimlab.github.io/hisat2/, accessed on 10 April 2025) to obtain mapped reads for subsequent analysis. FeatureCounts (v2.1.1) was used to count the reads mapped to each gene, and FPKM values for each gene were calculated based on gene length. Differentially expressed genes (DEGs) between transgenic Arabidopsis and WT plants were identified using DESeq2 based on the default thresholds of |log2FoldChange| > 1 and *p*-value (*p*adj) < 0.05 [30]. GO and KEGG enrichment analyses of DEGs were performed using the clusterProfiler R package [31]. Terms with a *p*-value < 0.05 were considered significantly enriched.

### 2.10. Metabolomics Analysis

Analyses were performed using a UHPLC system (1290 Infinity LC, Agilent Technologies, Santa Clara, CA, USA) coupled to a quadrupole time-of-flight mass spectrometer (AB Sciex TripleTOF 6600) at Shanghai Applied Protein Technology Co., Ltd., Shanghai, China. The raw MS data (wiff.scan files) were converted to MzXML files using MSConvert (ProteoWizard, v3.0.23131) before importing into the freely available MS-DIAL software [32]. For peak picking, the following parameters were used: centWave m/z = 10 ppm, peakwidth = c (10, 60), and prefilter = c (10, 100). For peak grouping, bw = 5, mzwid = 0.025, and minfrac = 0.5 were used. CAMERA (Collection of Algorithms of MEtabolite pRofile Annotation) was used for the annotation of isotopes and adducts. In the extracted ion features, only variables having more than 50% of non-zero measurement values in at least one group were retained. Metabolite identification was performed by comparing the accurate m/z value (<10 ppm) and MS/MS spectra with an in-house database established using available authentic standards. After normalization to total peak intensity, the processed data were analyzed using the R package ‘ropls’, undergoing multivariate data analysis, including Pareto-scaled principal component analysis (PCA) and orthogonal partial least squares discriminant analysis (OPLS-DA). The robustness of the model was evaluated using 7-fold cross-validation and response permutation testing. The variable importance in the projection (VIP) value of each variable in the OPLS-DA model was calculated to indicate its contribution to the classification. Metabolites with VIP values >1 were further subjected to Student’s *t*-test at the univariate level to measure the significance of each metabolite; *p*-values less than 0.05 were considered statistically significant.

## 3. Results

### 3.1. Correlation Analysis Between the Expression Level of the EuGT8 Gene and the PDG Contents

**Figure 1:**
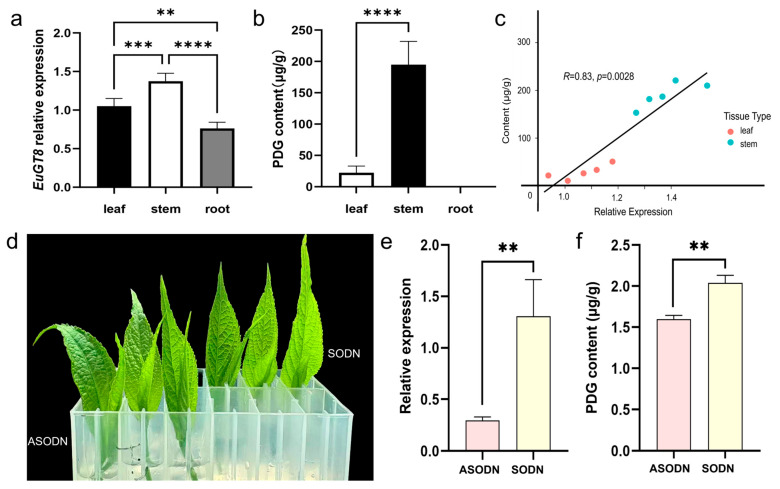
Determination of the expression levels of the *EuGT8* gene and PDG contents in three different organs of *E. ulmoides*. (**a**) The expression patterns of the *EuGT8* gene in three different organs of *E. ulmoides*. (**b**) The PDG contents in three different organs of *E. ulmoides*. (**c**) Correlation analysis between the expression level of the *EuGT8* gene and the PDG contents. (**d**) Apical buds of *E. ulmoides* treated with asODN targeting *EuGT8* gene. (**e**) The expression levels of the *EuGT8* gene in apical buds of *E. ulmoides* treated with asODN. (**f**) The PDG contents in apical buds of *E. ulmoides* treated with asODN. Note: ** *p* < 0.01, *** *p* < 0.001, and **** *p* < 0.0001.

Based on our laboratory’s transcriptome data, a candidate glycosyltransferase gene named *EuGT8* was isolated and identified. We also amplified the *EuGT8* gene by high-fidelity PCR using specific primers and analyzed the sequence variations (Figure S1a,b). Results showed that the cloned sequence was consistent with the genomic sequence. To explore the gene function of *EuGT8* in regulating the PDG biosynthesis, we further investigated the expression pattern of the *EuGT8* gene and the dynamic changes in PDG in three different organs of *E. ulmoides* (Figure 1a,b). Results showed that the expression level of the *EuGT8* gene in aerial parts was significantly higher than that in roots, and its expression in stems was higher than that in leaves (Figure 1a). The PDG contents were only detected in the aerial parts of *E. ulmoides*, and the PDG contents in stems were higher than those in leaves (Figure 1b). Correlation analysis revealed that the expression level of the *EuGT8* gene and PDG contents exhibited a strong positive correlation (Figure 1c). These results suggested that the *EuGT8* gene might play an important role in regulating PDG biosynthesis.

To further substantiate the *EuGT8* gene’s involvement in PDG biosynthesis in *E. ulmoides*, we utilized asOND targeting the *EuGT8* gene to one bud with two leaves. Results demonstrated that the expression level of the *EuGT8* gene and PDG contents in one bud with two leaves treated with asODN exhibited a significant reduction relative to the control group (sODN) (Figure 1d,e). These results suggested that the *EuGT8* gene might be associated with the PDG biosynthesis in *E. ulmoides*.

### 3.2. Subcellular Localization and Enzymatic Activity of EuGT8 Gene

**Figure 2:**
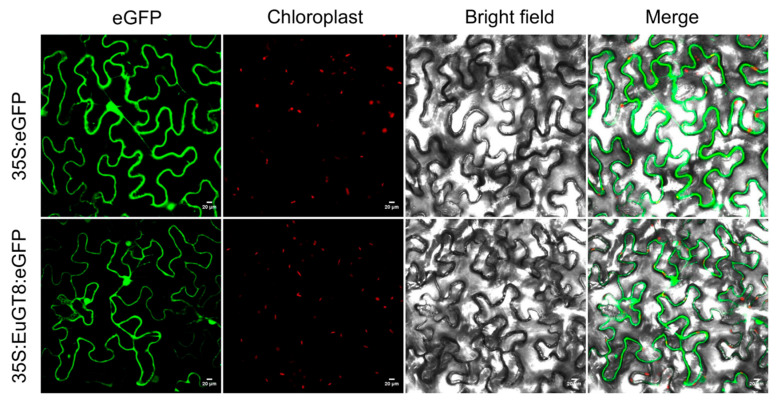
Subcellular distribution of EuGT8 protein. GFP alone or GFP fusions with EuGT8, driven by the CaMV35S promoter, were transiently expressed in tobacco leaf epidermal cells. The localization of the fusion proteins was visualized using laser scanning confocal microscopy. Scale bar = 20 µm.

To further investigate the gene function of the *EuGT8* gene, we determined the subcellular localization of the *EuGT8* gene in tobacco leaves via transient transformation. The fluorescence results of infected tobacco leaves revealed that the EuGT8-GFP fusion protein was located in the cytoplasm (Figure 2), whereas free GFP was detected throughout the cell. This result indicated that the *EuGT8* gene likely functions in the cytoplasm.

**Figure 3:**
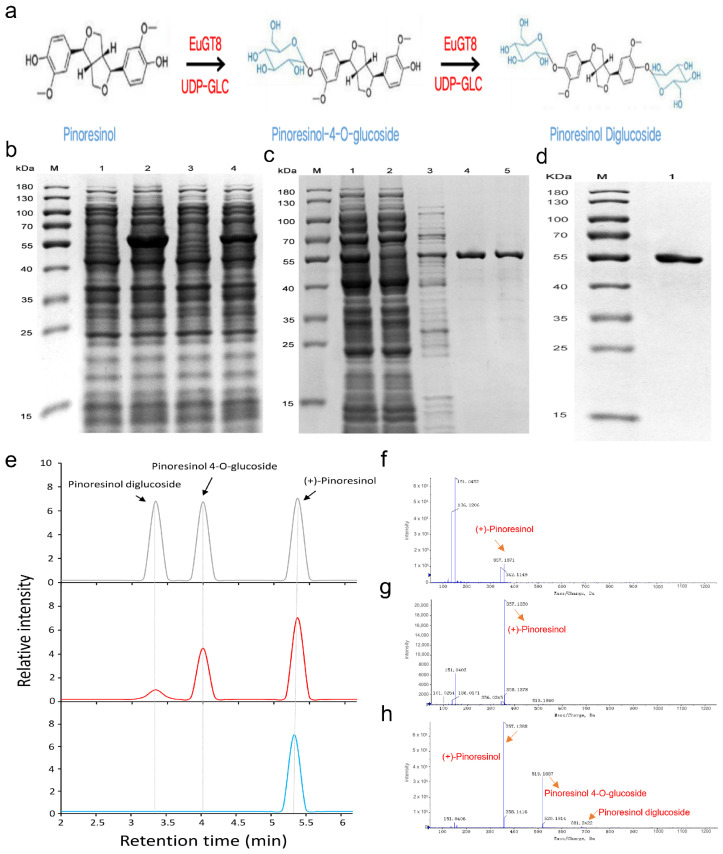
The prokaryotic expression of the *EuGT8* gene. (**a**) The two-step glycosylation of pinoresinol produced pinoresinol-4-O-glucoside, followed by pinoresinol diglucoside. (**b**) Protein expression analysis. (**c**) Protein purification analysis. (**d**) Protein dialysis analysis. (**e**) HPLC analysis of the products of the reactions catalyzed by the EuGT8 using pinoresinol as the substrate. Blue represents the negative control, red represents the reactions catalyzed by the EuGT8, and gray represents the positive control. (**f**–**h**) Ion fragmentation patterns of the substrate pinoresinol (**f**), pinoresinol 4-O-glucoside (**g**), and pinoresinol diglucoside (**h**) were further confirmed using LC-MS/MS. The reaction product PDG shows a major fragment ion peak at m/z 681.2 (M+2Glc−H+) with fragment ions at m/z 519.2 (M+Glc−H+), m/z 357.2 (M−H+), and m/z 161.0. (**e**) The two-step glycosylation of pinoresinol, producing pinoresinol-4-O-glucoside, followed by pinoresinol diglucoside.

The PDG, as an important lignan compound, was mainly detected in the bark of *E. ulmoides*, which was generated via the glycosylation of pinoresinol (Figure 3a). Thus, we obtained EuGT8 protein via prokaryotic expression and performed in vitro reactions using pinoresinol as the substrate and UDP-glucose as the sugar donor (Figure 3b–d). Analysis of the reaction mixture by UPLC-MS/MS revealed the generation of two compounds from pinoresinol in the presence of EuGT8 (Figure 3e–h). Mass spectrometric analysis identified these as pinoresinol-4-O-glucoside and pinoresinol diglucoside (Figure 3e–h). This indicates that EuGT8 can utilize both pinoresinol and pinoresinol-4-O-glucoside as substrates, with UDP-glucose as the sugar donor (Figure 3f), to glycosylate them into pinoresinol-4-O-glucoside (Figure 3g) and pinoresinol diglucoside, respectively (Figure 3h). The content of pinoresinol-4-O-glucoside in the reaction mixture was much higher than that of pinoresinol diglucoside (Figure 3h), suggesting that EuGT8 glycosylates pinoresinol more efficiently than pinoresinol-4-O-glucoside.

### 3.3. Generated Transgenic Arabidopsis Overexpressing EuGT8 Gene

**Figure 4:**
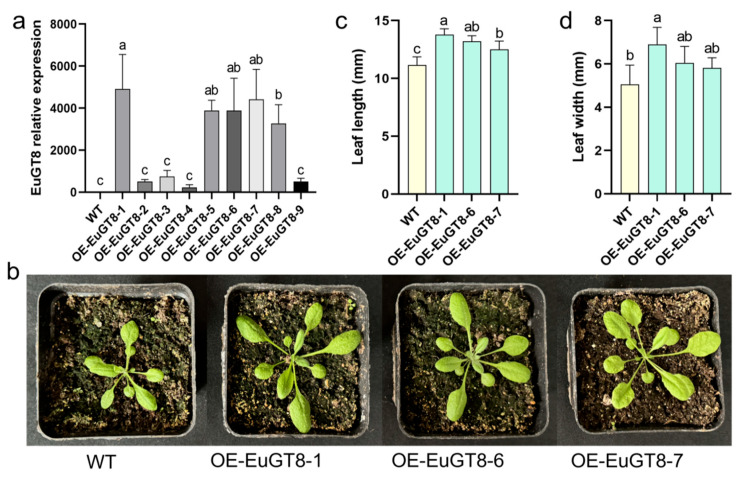
Generation of transgenic *Arabidopsis* lines overexpressing the *EuGT8* gene. (**a**) The expression levels of the *EuGT8* gene in nine transgenic *Arabidopsis* lines. (**b**) Phenotypic characteristics of transgenic *Arabidopsis* lines overexpressing the *EuGT8* gene. (**c**,**d**) Determination of the leaf length (**c**) and leaf width (**d**) of transgenic *Arabidopsis* and WT plants. The data are the mean  ±  SE of three independent biological samples. Lowercase letters indicate significant differences between treatments (*p* < 0.05).

To investigate the molecular mechanism of the *EuGT8* gene involved in PDG biosynthesis in *E. ulmoides*, we generated the transgenic *Arabidopsis* lines overexpressing the *EuGT8* gene using the floral dip transformation method. A total of nine transgenic *Arabidopsis* lines were obtained based on the PCR verification and hygromycin selection (Figure 4a and Figure S1c). Among them, three T_3_ homozygous lines (OE-EuGT8-1, OE-EuGT8-6, and OE-EuGT8-7) with high expression levels of *EuGT8* were selected to perform phenotypic observation, as well as integrated transcriptomic and metabolomic analysis (Figure 4). Results showed that the size of the transgenic *Arabidopsis* plants was significantly larger than that of wild-type (WT) plants (Figure 4b). Further, the leaf length and leaf width in EuGT8-OE lines were significantly increased by 11.60–12.97% and 4.94–6.17% compared with WT plants, respectively (Figure 4c,d). These results indicated that the *EuGT8* gene played an essential role in promoting the growth and development of *E. ulmoides*.

### 3.4. Transcriptomic Analysis of Transgenic Arabidopsis and WT Plants

**Figure 5:**
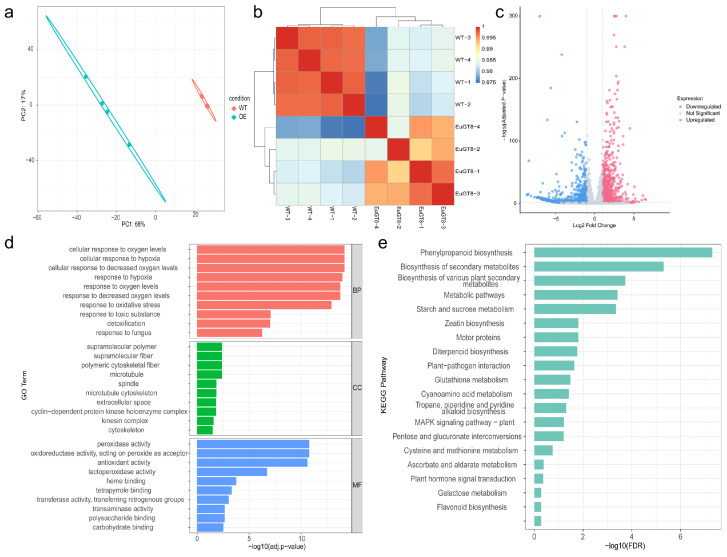
Transcriptomic analysis of transgenic *Arabidopsis* overexpressing *EuGT8* and WT plants. (**a**) PCA. (**b**) Hierarchical clustering heatmap of DEGs. (**c**) Volcano plot of gene expression changes. (**d**) GO enrichment analysis of DEGs. (**e**) KEGG pathway enrichment analysis of DEGs.

To further explore the molecular regulatory mechanism of the *EuGT8* gene, we performed transcriptomic analysis of transgenic *Arabidopsis* and WT plants (Figure 5). The PCA result revealed a distinct separation between transgenic *Arabidopsis* and WT plants, indicating that the transcriptome possessed excellent reproducibility and reliability (Figure 5a). The strong correlations detected in the different biological replicates in each group indicated that the dataset was both consistent and reliable for subsequent analyses (Figure 5b). According to the screening criteria of FDR < 0.05 and |Log2FC| ≥ 1, a total of 1799 DEGs (774 up- and 1025 down-regulated) were identified in OE-EuGT8-1 vs. WT plants (Figure 5c, Table S2). GO analysis revealed that the DEGs were mainly enriched in three main functional categories: molecular function (MF), cellular component (CC), and biological process (BP) (Figure 5d, Table S3). In the MF category, the DEGs were mainly enriched in peroxidase activity, oxidoreductase activity, acting on peroxide as acceptor, antioxidant activity, lactoperoxidase activity, and so on. In the CC category, the DEGs were mainly enriched in supramolecular polymer, supramolecular fiber, polymeric cytoskeletal fiber, microtubule, and so on. In the BP category, the DEGs were mainly enriched in cellular response to oxygen levels, cellular response to hypoxia, cellular response to decreased oxygen levels, and response to hypoxia. To further investigate the DEGs involved in the metabolic pathway, we performed KEGG enrichment analysis of the DEGs between transgenic *Arabidopsis* and WT plants (Figure 5e). Results showed that the DEGs were mainly enriched in phenylpropanoid biosynthesis (30 genes), secondary metabolite biosynthesis (138 genes), and starch/sucrose metabolism (25 genes) pathways (Figure 3d).

### 3.5. Validation of Transcriptome Data Using RT-qPCR

**Figure 6:**
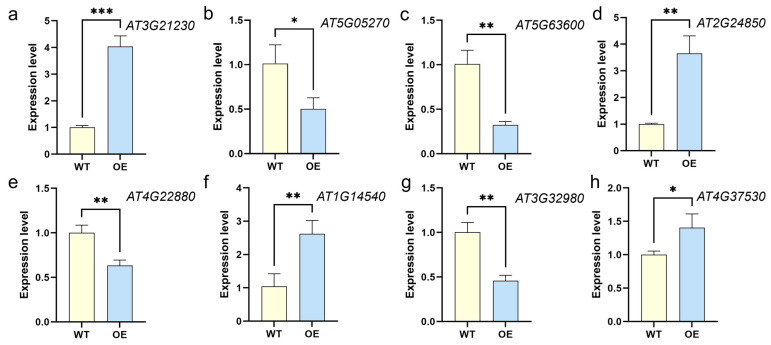
Expression levels of 8 selected DEGs in transgenic *Arabidopsis* plants. (**a**) *AT3G21230*; (**b**) *AT5G05270*; (**c**) *AT5G63600*; (**d**) *AT2G24850*; (**e**) *AT4G22880*; (**f**) *AT1G14540*; (**g**) *AT3G32980*; (**h**) *AT4G37530*. Total RNA was extracted from transgenic *Arabidopsis* (OE) and WT plants, and was used to perform RT-qPCR analysis with ACTING gene as the housekeeping gene. The 2^−ΔΔCt^ method was applied to the expression levels of each candidate. Note: * *p* < 0.05, ** *p* < 0.01 and *** *p* < 0.001.

To validate the RNA-seq results, eight DEGs between *Arabidopsis* and WT plants were selected and verified by using RT-qPCR (Figure 6). Results showed that the expression trends of eight selected candidate DEGs in transgenic *Arabidopsis* plants, as shown by RT-qPCR results, were consistent with the RNA-Seq data, supporting the reliability of the transcriptome data.

### 3.6. Metabolomic Analysis of Transgenic Arabidopsis and WT Plants

**Figure 7:**
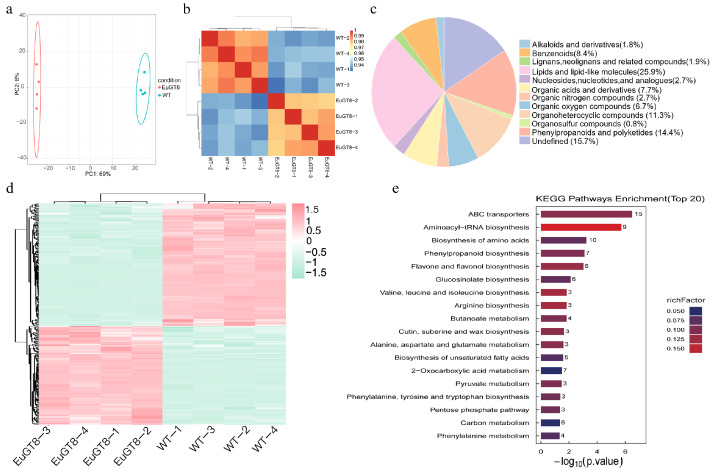
Metabolomic analysis of transgenic *Arabidopsis* and WT plants. (**a**) PCA of metabolites. (**b**) Hierarchical clustering heatmap of differential metabolites. (**c**) KEGG pathway enrichment analysis of differential metabolites (Top 20). (**e**) The correlation heatmap of top 50 metabolites. (**d**) Classification of metabolite classes (pie chart).

To investigate the impact of *EuGT8* on the biosynthesis of secondary metabolites, we conducted the metabolomic analysis on transgenic *Arabidopsis* overexpressing *EuGT8* (Figure 7). Eighteen distinct chemical categories encompassed the 960 metabolites detected in this analysis. The results of PCA revealed that transgenic *Arabidopsis* and WT samples were distinctly separated according to PC1 and PC2 (Figure 7a), indicating distinct metabolite compositions between the transgenic *Arabidopsis* and WT plants. The strong correlations observed among the biological replicates in every group indicated that the dataset was both consistent and reliable for subsequent analyses (Figure 7b). Among them, lipids and lipid-like molecules represented the largest group (231, 25.9%), followed by phenylpropanoids and polyketides (129, 14.4%), organoheterocyclic compounds (101, 11.3%), benzenoids (75, 8.4%), organic acids and derivatives (69, 7.7%), organic oxygen compounds (60, 6.7%) and so on (Figure 7c).

Applying the rigorous thresholds of FDR < 0.05 and |Log2FC| ≥ 1.5, a total of 294 DEMs (131 up- and 163 down-regulated) were identified (Figure 7d, Table S4). Enrichment analysis of the DEMs in KEGG pathways showed that the DEMs were enriched in 68 distinct pathways. Among the top 20 KEGG pathways, the DEMs were mainly enriched in six KEGG pathways, including ABC transporters, aminoacyl-tRNA biosynthesis, biosynthesis of amino acids, phenylpropanoid biosynthesis, flavone and flavonol biosynthesis, and glucosinolate biosynthesis (Figure 7e, Table S5).

### 3.7. Correlation Analysis Between Transcriptome and Metabolome

**Figure 8:**
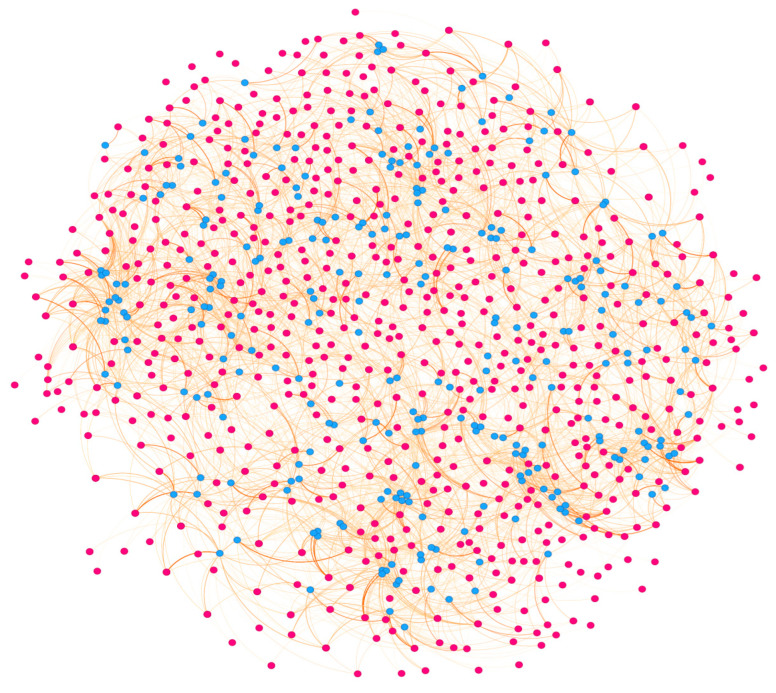
Correlation network of DEMs and DEGs in transgenic *Arabidopsis* and WT plants. The red and blue solid circles represent the DEGs and DEMs, respectively. The orange line represents the correlation (r > 0.95).

To further investigate the regulatory mechanism of DEGs in metabolite biosynthesis, we performed a correlation analysis between DEGs and DEMs in transgenic *Arabidopsis* plants in comparison with WT plants, selecting those with Pearson correlation coefficients exceeding 0.95 (Figure 8, Table S6). The results showed that a total of 231 DEGs associated with 38 DEMs were identified, mainly including 24 DEGs associated with four DEMs in the phenylpropanoid biosynthesis pathway, 24 DEGs associated with one DEM in starch and sucrose metabolism, 12 DEGs associated with three DEMs in the biosynthesis of amino acids, three DEGs associated with two DEMs in flavonoid biosynthesis, three DEGs associated with one DEM in phenylalanine, tyrosine, and tryptophan biosynthesis, and two DEGs associated with one DEM in phenylalanine metabolism. These results suggested that the *EuGT8* gene might be associated with the dynamic changes in metabolites involved in multiple metabolic pathways.

## 4. Discussion

Plant glycosyltransferases constitute a large multigene superfamily that mediates the transfer of activated mono- or oligosaccharides to various acceptor molecules, ultimately leading to the glycosylation of plant compounds [12,13,14,33]. Although the identification of the genes encoding glycosyltransferases has been conducted in many plants, only a few genes of this family have been functionally characterized [34,35]. However, the genes encoding glycosyltransferases associated with lignan glycosylation in *E. ulmoides* have not been reported to date. The PDG, as important lignan compounds, were mainly detected in the bark of *E. ulmoides*, which were generated via the glycosylation of pinoresinol [34,36,37]. In this study, we screened and cloned the *EuGT8* gene from *E. ulmoides* based on our transcriptome data. The expression pattern of the *EuGT8* gene exhibited a strong positive correlation with dynamic changes in the PDG contents. The expression levels of the *EuGT8* gene and PDG contents were significantly decreased and increased in asODN-*EuGT8*-treated shoot tips of *E. ulmoides* in comparison with the control group. These results indicated that the *EuGT8* gene might play an important role in regulating the PDG biosynthesis

It is well known that the elucidation of the subcellular localization of proteins is very important in order to deeply understand their functions [38,39]. In the present study, our results showed that the EuGT8-GFP fusion protein was located in the cytoplasm, indicating that the *EuGT8* gene likely functions in the cytoplasm. Moreover, the prokaryotic expression analysis was applied to identify and functionally characterize the target gene related to the biosynthesis of secondary metabolites. Here, prokaryotic expression of the *EuGT8* gene revealed that the purified EuGT8 protein could catalyze the conversion of pinoresinol into pinoresinol-4-O-glucoside and PDG, and the PDG content was lower than that of pinoresinol-4-O-glucoside. These results indicated that the *EuGT8* gene was involved in mediating the biosynthesis of PDG and the corresponding intermediate products, which is consistent with the previous studies that found that the genes encoding glycosyltransferases were involved in regulating lignan glycosylation [17,18,33,40]. For instance, tandem *UGT71B5* genes in *I. indigotica* have been reported to participate in catalyzing lignan glycosylation [17]. In *L. usitatissimum*, the *LuUGT74S1* gene has been reported to control the formation of secoisolariciresinol diglucoside [18,41,42].

In addition, to further investigate the broad-spectrum activity of the *EuGT8* gene in regulating the glycosylation of secondary metabolites, we generated nine transgenic *Arabidopsis* lines overexpressing the *EuGT8* gene. Results revealed that the leaf length and leaf width in EuGT8-OE lines were significantly larger than those in WT plants, demonstrating that the *EuGT8* gene might be involved in the regulation of leaf development. This result was consistent with the previous studies reporting that glycosyltransferase-encoding genes were involved in mediating plant growth and development [43,44,45,46]. Transcriptomic analysis of transgenic *Arabidopsis* and WT plants showed that a total of 1799 DEGs were identified in EuGT8-overexpressing *Arabidopsis* compared to WT plants. KEGG enrichment analysis revealed that the DEGs were primarily enriched in several metabolic pathways, such as phenylpropanoid biosynthesis, secondary metabolite biosynthesis, starch and sucrose metabolism, and so on. Untargeted metabolomic analysis of OE-EuGT8 *Arabidopsis* and WT plants revealed that the contents of phenylpropanoids, flavonoids, and glycosides were significantly increased in transgenic *Arabidopsis* in comparison with WT plants. Both transcriptomic and metabolomic results indicate that EuGT8 plays a significant role in the biosynthesis of secondary metabolites, particularly phenylpropanoids and flavonoids.

## 5. Conclusions

PDG, one of the major lignans isolated from *E. ulmoides*, has various pharmacological functions, including antihypertension effects and prevention of osteoporosis. In this study, we screened and cloned the *EuGT8* gene from *E. ulmoides*. The expression pattern of the *EuGT8* gene exhibited a strong positive correlation with the dynamic changes in the PDG contents. The expression level of the *EuGT8* gene and PDG contents were significantly decreased in asODN-*EuGT8*-treated shoot tips in comparison with the control group. Prokaryotic expression of the *EuGT8* gene revealed that the purified EuGT8 protein could catalyze the conversion of pinoresinol into PDG. Additionally, we generated transgenic *Arabidopsis* lines overexpressing the *EuGT8* gene and performed transcriptional and metabolomic analyses on both transgenic and WT plants. The results showed that a total of 1799 DEGs and 294 DEMs were identified between transgenic and WT plants. Correlation analysis between DEGs and DEMs identified a total of 231 DEGs associated with 38 DEMs, which were mainly distributed in phenylpropanoid biosynthesis, starch and sucrose metabolism, biosynthesis of amino acids, flavonoid biosynthesis, and phenylalanine, tyrosine, and tryptophan biosynthesis pathways. Collectively, these results suggest that *EuGT8* is likely involved in the glycosylation of lignans and the regulation of multiple metabolic pathways, thus providing a basis for further exploration of the biological functions of plant glycosyltransferases.

## Data Availability

Data will be made available on request.

## Supplementary Materials

The following supporting information can be downloaded at: https://www.mdpi.com/article/10.3390/life16040622/s1; Figure S1. Cloning, alignment, and validation of transgenic plants for the EuGT8 gene. Table S1. The primers involved in experiment and their functions. Table S2. DEGs in the transcriptome of WT and OE plants. Table S3. GO analysis of differentially expressed genes in the transcriptome. Table S4. Differentially expressed genes in the metabolome. Table S5. Annotation information of the top 20 differential metabolites in the metabolome KEGG enrichment analysis. Table S6. Correlation analysis between transcriptome and metabolome.
