# Supplementary material for: Identification of a Glycosyltransferase-Encoding Gene (EuGT8) from Eucommia ulmoides That Catalyzes the Glycosylation of Pinoresinol to Pinoresinol Diglucoside
Source: Life (Basel). 2026 Apr 8;16(4):622. doi: 10.3390/life16040622 (PMC13117410; doi:10.3390/life16040622)
Supplement: Supplementary file 1 [file life-16-00622-s001.zip › Supplementary Figure.pdf]

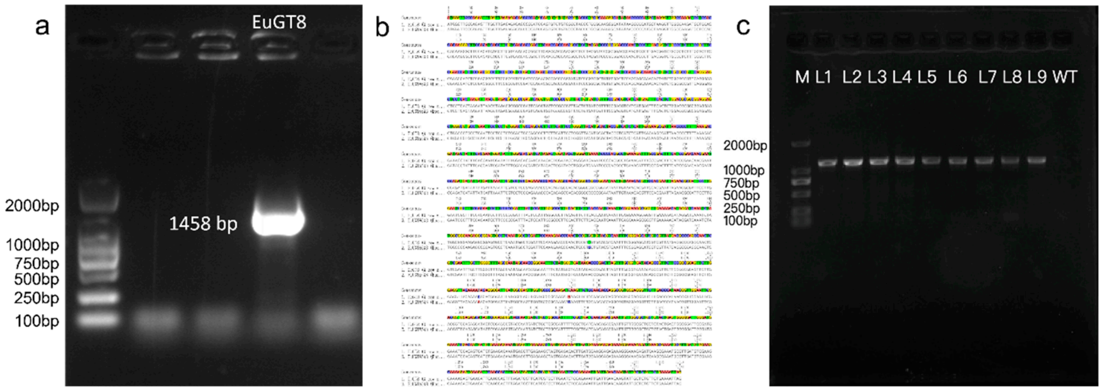

Figure S1 Cloning, alignment, and validation of transgenic plants for the EuGT8 gene. (a)Cloning of the *EuGT8* gene. M: DNA marker. (b) Alignment of the cloned sequence with the genomic sequence. (c) PCR validation of *EuGT8* transgenic plants. WT: wild-type *Arabidopsis*; L1–L9: nine transgenic lines.
